# Optimizing diagnostic outcomes and sustainability in radiological practices: a multicentric study on guideline adherence and incidental findings in elective chest CT scans

**DOI:** 10.1007/s11547-025-02133-5

**Published:** 2025-10-27

**Authors:** Jacopo D’Argenzio, Andrea Esposito, Jacopo Pozzi, Caterina Giannitto, Massimo Zilocchi, Matteo Stocco, Marzia Guerritore, Giuseppina Maria Rita Valenti, Mario Giovanni Melazzini, Gianpaolo Carrafiello

**Affiliations:** 1 Postgraduate School in Radiodiagnostics, Università Degli Studi Di Milano, Via Festa del Perdono 7, 20122 Milan, Italy; 2 Department of Diagnostic Radiology, ASST Bergamo Ovest, Treviglio-Caravaggio Hospital, Piazzale Ospedale 1, 24047 Treviglio, BG Italy; 3 Department of Diagnostic and Interventional Radiology, IRCCS Humanitas Research Hospital, Via Manzoni 56, 20089 Rozzano, MI Italy; 4 Department of Biomedical Sciences, Humanitas University, Via Rita Levi Montalcini 4, 20072 Pieve Emanuele, MI Italy; 5 Radiology Department, Fondazione IRCCS Cà Granda Ospedale Maggiore Policlinico, Via Francesco Sforza 35, 20122 Milan, Italy; 6 General Management, Fondazione IRCCS Ca’ Granda Ospedale Maggiore Policlinico, Via Francesco Sforza 35, 20122 Milan, Italy; 7 Organizational Unit for Service Provision and Cross-Cutting Projects, Directorate General for Welfare, Lombardy Region, Piazza Città Di Lombardia 1, 20124 Milan, Italy; 8 Directorate General for Welfare, Lombardy Region, Piazza Città Di Lombardia 1, 20124 Milan, Italy; 9 Department of Oncology and Hematology-Oncology, Università Degli Studi Di Milano, Via Festa del Perdono 7, 20122 Milan, Italy

**Keywords:** Chest CT, Incidental findings, Guideline adherence, Healthcare sustainability, Radiological recommendations

## Abstract

**Purpose:**

This multicentric retrospective study aimed to evaluate the diagnostic outcomes, adherence to guideline-based recommendations, and sustainability implications of 280 initial chest CT scans performed without contrast. The scans were conducted at the Radiology Unit of the Foundation IRCCS Ca’ Granda Ospedale Maggiore Policlinico of Milan and the Radiology Department of Treviglio-Caravaggio Hospital-ASST Bergamo Ovest. The study focused on optimizing patient selection and radiological recommendations to align with evidence-based guidelines.

**Materials and methods:**

This retrospective study included 280 patients (mean age 61.68 years; 51.07% women, 48.93% men) who underwent their first chest CT scan without contrast. Diagnostic outcomes were analyzed across different clinical queries. Incidental findings and radiologists’ recommendations were assessed for alignment with Fleischner Society and ACR guidelines. Deviations were categorized as unnecessary imaging suggestions or missed indications. Radiation exposure was quantified using the dose-length product (DLP).

**Results:**

Diagnostic outcomes were positive in 54.64% of cases. Incidental findings occurred in 34.64% of cases, with guideline adherence at 81.43%. Deviations included unnecessary imaging in 14.29% and missed follow-up indications in 4.28% of cases. The median DLP was 237.5 mGy·cm (IQR 171.8–320.1).

**Conclusion:**

This study highlights significant opportunities to refine patient selection and radiological recommendations through adherence to Fleischner Society and ACR guidelines. By integrating evidence-based practices into routine workflows, the findings advocate for reduced unnecessary imaging, enhanced diagnostic pathways, and sustainable healthcare practices in chest CT imaging.

## Introduction

Computed tomography (CT) has become an indispensable tool in diagnostic imaging, offering unparalleled resolution and cross-sectional visualization of anatomical structures. Its utility spans a wide array of clinical scenarios, ranging from acute pathologies to routine assessments of chronic conditions. However, the increasing reliance on CT imaging raises significant concerns regarding the balance between its diagnostic benefits and the risks associated with ionizing radiation, particularly in non-critical and elective settings. The challenge lies in optimizing patient selection and ensuring that imaging contributes meaningfully to clinical decision-making while minimizing unnecessary exposure and healthcare resource utilization.

In elective imaging scenarios, where clinical urgency is absent, the need for stringent preselection criteria becomes paramount. This is particularly relevant for patients undergoing their first CT scan, as they often represent cases where careful clinical evaluation could potentially obviate the need for imaging. Additionally, CT imaging frequently captures structures beyond the primary anatomical region of interest, leading to the detection of incidental findings [1]. While such findings can offer valuable diagnostic opportunities, they also pose challenges related to follow-up recommendations, potentially leading to unnecessary investigations or overlooked indications if guideline adherence is not rigorously maintained. These findings highlight opportunities to improve reporting consistency and guideline-based follow-up, supporting more sustainable imaging practices.

This retrospective, multicentric study investigates 280 initial CT scans performed on adult patients across two major radiology departments in Italy: the Foundation IRCCS Ca’ Granda Ospedale Maggiore Policlinico Hospital of Milan and the Treviglio-Caravaggio Hospital-ASST Bergamo Ovest. All scans were conducted in elective settings, excluding emergency and urgent cases, with patients carefully selected to ensure that no prior CT imaging had been performed. The distribution of cases across the calendar year further mitigates potential biases associated with seasonal variations in disease prevalence.

The primary objectives of the study were threefold. First, to evaluate the diagnostic outcomes of these initial CT scans, including the proportion of cases where imaging yielded findings consistent with the clinical diagnostic query. Second, to assess the prevalence and management of incidental findings, with a particular focus on their alignment with established guidelines, such as those provided by the Fleischner Society [2] and the American College of Radiology (ACR) [3–10], which offer comprehensive guidance on pulmonary nodules, thoracic findings, and incidental extra-pulmonary findings. Third, to explore the broader implications of imaging practices on healthcare sustainability, considering not only the potential for radiation overexposure but also the downstream impact of non-evidence-based recommendations.

Importantly, while most scans focused on the thoracic region, this study also accounts for findings in anatomically adjacent areas, such as the thyroid gland, liver, and kidneys, which were included within the imaging field of view. These ‘extra-thoracic’ findings require consistent classification and guideline-based follow-up recommendations across organ systems.

By integrating real-world data from two distinct institutions, this study sheds light on opportunities to refine imaging pathways, improve adherence to evidence-based guidelines, and enhance the sustainability of radiological practice. Radiology services can support appropriate use by applying guideline-based recommendations and standardized follow-up: aligning practice with established guidelines is key to achieving these goals, paving the way for a more resource-conscious and diagnostically effective healthcare system.

## Materials and methods

This observational retrospective study included a cohort of 280 chest CT scans conducted without contrast enhancement, performed between January and December 2023 at two radiology departments in Northern Italy: the Foundation IRCCS Ca’ Granda Ospedale Maggiore Policlinico Hospital of Milan (200 cases) and the Treviglio-Caravaggio Hospital (80 cases). The study was designed to evaluate diagnostic outcomes, incidental findings, and adherence to evidence-based guidelines in the management of radiological findings.

### Study design and inclusion criteria

The selection process adhered to strict inclusion criteria to ensure consistency and focus. Only adult patients (aged ≥ 18 years) undergoing their first chest CT scan were considered, with no prior history of computed tomography, except for plain radiographs, if applicable. Patients with known oncological pathologies or those undergoing CT in emergency or urgent care settings were excluded. This deliberate restriction to elective CT scans facilitated a more precise evaluation of diagnostic appropriateness and guideline adherence under non-acute conditions.

To limit seasonal bias, scans were evenly distributed across the calendar year. This reduced the impact of conditions with temporal trends that might otherwise confound results, such as respiratory infections or inflammatory disorders.

Additionally, the distribution reflects routine clinical practice across different times of the year, enhancing the generalizability of findings.

### Patient demographics and baseline characteristics

The final cohort comprised 143 females (51.07%) and 137 males (48.93%), with a mean age of 61.68 years (range 18–92 years). These demographic parameters reflect a balanced population sample, representative of patients typically undergoing elective chest CT in the participating institutions.

### Data collection and imaging protocol

In this multicentric retrospective study, chest CT scans were acquired by certified radiographers according to standardized non-contrast protocols, optimized for thoracic imaging. At Treviglio-Caravaggio Hospital, all scans were acquired using a Toshiba Aquilion CT scanner (Canon Medical Systems Europe), with an acquisition protocol set at 120 kVp. At the Foundation IRCCS Ca’ Granda Ospedale Maggiore Policlinico Hospital of Milan, CT examinations were conducted using a Siemens SOMATOM X.cite (Siemens Healthineers), also employing an acquisition protocol of 120 kVp.

All scans were systematically acquired from the lung apices to the bases using standardized imaging protocols to ensure consistency across institutions. While the primary focus remained on thoracic structures, scan coverage in some cases extended to adjacent anatomical regions, such as the upper abdomen (liver, kidneys) and the thyroid gland, depending on patient positioning and individual acquisition parameters. This approach facilitated a thorough assessment of primary diagnostic findings while also allowing for the identification of incidental discoveries.

All images were independently reviewed by two board-certified radiologists, each with over 15 years of experience in thoracic imaging, at their respective institutions. Standard pulmonary and mediastinal reconstructions were assessed, with a slice thickness of 2 mm applied uniformly across both sites to ensure optimal evaluation of lung parenchyma, mediastinal structures, and incidental findings.

Diagnostic outcomes were categorized as positive or negative based on the primary clinical query. Incidental findings, defined as unexpected radiological abnormalities unrelated to the clinical query, were separately documented. Radiological recommendations for follow-up or additional investigations were assessed for their alignment with established guidelines, including the Fleischner Society recommendations for pulmonary nodules and the American College of Radiology (ACR) guidelines for incidental findings in the abdomen⁷ and thyroid.

The decision to rely primarily on Fleischner Society and ACR guidelines stems from the absence of equivalent European guidelines addressing incidental findings in certain organ systems, such as the liver, in the context of CT imaging. Current European guidelines, including those endorsed by the European Society of Radiology (ESR), primarily focus on ultrasonographic evaluation of liver abnormalities and do not comprehensively cover incidental findings on CT. For this reason, the Fleischner Society and ACR guidelines, which are internationally recognized and widely adopted in clinical practice, were deemed the most appropriate references for this study. These guidelines provide robust and evidence-based recommendations that address incidental findings across multiple organ systems and imaging modalities.

European guidance on incidental findings is heterogeneous and mostly program- or organ-specific (e.g., LDCT lung cancer screening statement) [11]. In the absence of a pan-European, radiology-led, cross-modality compendium comparable to the ACR Incidental Findings Committee corpus, we used ACR Incidental Findings and Fleischner recommendations to ensure consistent management.

### Outcome measures and data analysis

The study evaluated three primary outcomes:

1. *Diagnostic yield*: The proportion of scans yielding findings relevant to the primary clinical query.
2. *Incidental findings*: The prevalence and types of incidental findings, including their anatomical distribution and management.
3. *Guideline adherence*: The extent to which radiological recommendations for incidental findings aligned with guideline-based practices.

Radiological recommendations were classified into categories, such as additional imaging (e.g., follow-up CT, ultrasound, or MRI), clinical evaluation, laboratory correlation, or no further action. Non-adherence was defined as either unwarranted recommendations (suggesting unnecessary tests) or failure to recommend appropriate follow-up (overlooking actionable findings).

To ensure consistency, discrepancies between recommendations and guidelines were reviewed by a third radiologist. Cases with multiple recommendations were analyzed individually to assess the overall alignment for each prescribed action, ensuring an accurate evaluation of compliance.

### Statistical considerations

Data were analyzed descriptively, with frequencies and percentages calculated for categorical variables. Continuous variables, such as age and dose-length product (DLP), were reported as means with standard deviations. Subgroup analyses by diagnostic query and anatomical site of incidental findings were performed to identify patterns in diagnostic yield and guideline adherence.

By combining detailed imaging data with guideline-based evaluation, this study provides a comprehensive overview of current practices in elective chest CT imaging, offering actionable insights for improving diagnostic efficiency and sustainability.

Binomial confidence intervals (95% CI) for proportions were calculated using the Clopper-Pearson method. Comparisons of adherence rates between organ systems were performed using chi-squared tests. Continuous dose metrics (CTDIvol and DLP) were summarized as quartiles, median, interquartile range (IQR), mean and standard deviation, stratified by center and overall. Between-center differences in dose metrics were tested using the Mann–Whitney U test (location) and the Kolmogorov–Smirnov test (overall distribution). Two-sided *p* < 0.05 was considered statistically significant.

## Results

The overall diagnostic positivity rate across the 280 chest CT scans was 54.64% (153/280 cases). When stratified by clinical indication, the highest diagnostic yields were observed in patients with bronchiectasis, with 80.0% positivity (8/10; 95% CI 44.2–96.5), and in those presenting with hemoptysis, at 75.0% (6/8; 95% CI 40.9–92.9). Chronic respiratory conditions also showed a substantial diagnostic return, with a positivity rate of 66.7% (32/48; 95% CI 52.1–78.8), followed by patients with suspicious findings on chest X-ray, who exhibited a positivity rate of 63.9% (53/83; 95% CI 52.8–73.7). In cases with infectious or inflammatory clinical suspicion, the diagnostic yield was 58.8% (20/34; 95% CI 42.2–73.8). More moderate yields were recorded for patients categorized under “other” indications (47.1%, 8/17; 95% CI 26.2–69.0) and those with chronic cough (46.9%, 15/32; 95% CI 31.2–63.3). Lower rates of diagnostic positivity were seen in patients presenting with non-specific pain (40.0%, 4/10; 95% CI 16.8–68.7), systemic diseases (25.0%, 2/8; 95% CI 7.2–59.1), and dyspnea (22.2%, 4/18; 95% CI 8.9–45.2). Patients evaluated for asbestos exposure demonstrated a positivity rate of 50.0% (1/2; 95% CI 9.5–90.5), while no positive findings were reported in the group assessed for suspected pneumothorax (0/10; 95% CI 0.0–30.8).

These findings highlight the significant heterogeneity in diagnostic yield across clinical indications. High positivity rates in bronchiectasis, hemoptysis, chronic respiratory disorders, and radiographic abnormalities reaffirm the appropriateness of chest CT in these scenarios, where the imaging modality demonstrably contributes to clinical decision-making. Conversely, the limited diagnostic utility observed in cases of dyspnea, systemic diseases, and non-specific pain calls for more selective use of CT, guided by stringent clinical criteria. The absence of diagnostic findings in the pneumothorax cohort further suggests that CT may not represent the optimal first-line modality in such cases, emphasizing the need for context-specific imaging strategies (Table 1).

**Table 1:** Diagnostic query positivity rates

Diagnostic query	Total cases	Positive cases	Proportion (%)	95% CI (clopper–pearson)
Suspicious findings on X-Ray	83	53	63.9%	52.6%—74.1%
Chronic conditions	48	32	66.7%	51.6%—79.6%
Infectious-inflammatory conditions	34	20	58.8%	40.7%—75.4%
Cough	32	15	46.9%	29.1%—65.3%
Dyspnea	18	4	22.2%	6.4%—47.6%
Other	17	8	47.1%	23.0%—72.2%
Non-specific pain	10	4	40.0%	12.2%—73.8%
Pneumothorax	10	0	0.0%	0.0%—30.8%
Bronchiectasis	10	8	80.0%	44.4%—97.5%
Systemic diseases	8	2	25.0%	3.2%—65.1%
Hemoptysis	8	6	75.0%	34.9%—96.8%
Asbestos exposure	2	1	50.0%	1.3%—98.7%

Incidental findings were identified in 97 patients, accounting for 34.64% of the study population. Among these, 49 cases (17.50%) consisted exclusively of incidental findings, while 48 cases (17.14%) exhibited both incidental findings and findings relevant to the primary diagnostic query.

These findings spanned various anatomical regions within and adjacent to the thoracic cavity, including the thyroid, liver, and kidneys, reflecting the comprehensive nature of CT imaging.

Management of radiological findings, whether incidental or related to the primary diagnostic query, demonstrated an overall alignment with established guidelines in 81.43% (95% CI 76.4%—85.8%) of cases. Deviations from guideline-based recommendations were observed in 18.57% (95% CI 13.87%-23.24%), divided into two main categories: over-recommendations, where unnecessary follow-up imaging was suggested (14.29% 95% CI 10.4%—18.9%), and under-recommendations, where indicated follow-up actions were omitted (4.28% 95% CI 2.2%—7.4%). These findings underscore the importance of ensuring evidence-based practices to optimize the appropriateness and sustainability of radiological follow-up strategies.

Further analysis of guideline adherence by organ system revealed considerable variability. Pulmonary findings exhibited the highest adherence rate, at 87.12% (95% CI 81.57–91.15), significantly higher than those observed for thyroid (42.11%; 95% CI 24.86–61.84; *p* < 0.001), liver (36.36%; 95% CI 21.89–54.16; *p* < 0.001), and kidneys (40.00%; 95% CI 21.87–61.74; *p* < 0.001). The adherence rate for other incidental findings was 89.47% (95% CI 75.96–95.72), not significantly different from that of pulmonary findings (*p* = 0.66). These statistical comparisons were performed using the chi-square test for independence.

Lower adherence in non-pulmonary regions indicates scope for further standardization. The high adherence to guidelines in pulmonary findings likely reflects the widespread adoption of well-established recommendations such as those of the Fleischner Society, while the lower adherence for other anatomical districts may be due to more heterogeneous guidance or lack of clear protocols in radiological practice.

The study also analyzed the types of follow-up recommendations made by radiologists, highlighting variability in guideline adherence. Repeat CT imaging was recommended in 74 cases, of which 14 (18.9%) were deemed unnecessary according to established guidelines. Alternative imaging modalities were suggested in 42 cases, primarily ultrasonography (30 cases), followed by PET (10 cases) and MRI (2 cases). Among these, 27 recommendations (64.3%) were not aligned with guideline criteria, including 26 instances of ultrasonography and 1 instance of PET imaging being unnecessarily recommended. No inappropriate recommendations were observed for MRI.

Clinical evaluation was suggested in 23 cases, with deviations from guideline-based practice occurring in 2 instances (8.7%). These findings underscore the need for enhanced adherence to evidence-based recommendations, particularly in the use of alternative imaging modalities, where the overuse of ultrasonography represents a significant area for improvement. The high proportion of non-aligned recommendations in alternative imaging calls for targeted educational interventions to ensure consistent and judicious radiological practices (Tables 2 and 3).

**Table 2:** Recommendations by radiologists

Recommendation type	Total cases	Aligned with guidelines	Percentage aligned (%)	95% CI (clopper–pearson)
Follow-up with CT	74	60	81.1%	70.3%—89.3%
Alternative imaging	42	15	35.7%	21.6%—51.9%

**Table 3:** Breakdown of alternative imaging recommendations

Imaging modality	Total cases	Non-Aligned recommendations
Ultrasound	30	26
PET	10	1
MRI	2	0

Across the cohort, the median volume CT dose index was 6.08 mGy (IQR 4.42–8.84) and the median dose–length product was 237.5 mGy·cm (IQR 171.8–320.1). Both indices were consistently higher at the Radiology Department of Treviglio-Caravaggio Hospital—ASST Bergamo Ovest compared with the Radiology Unit of the Foundation IRCCS Ca’ Granda Ospedale Maggiore Policlinico of Milan (Table 4). For CTDIvol, Treviglio reported a median of 10.74 mGy (IQR 8.28–13.82), approximately twice the value observed at Policlinico (5.39 mGy, IQR 4.04–6.58). A similar trend was seen for DLP, with a median of 382.0 mGy·cm (IQR 282.6–466.4) at Treviglio versus 208.5 mGy·cm (IQR 158.8–254.0) at Policlinico. These differences reached statistical significance on both Mann–Whitney U and Kolmogorov–Smirnov testing (*p* < 0.0001 for both comparisons).

**Table 4:** CTDIvol and DLP by center and overall

Metric	Statistic	Foundation IRCCS Ca’ Granda Ospedale Maggiore Policlinico Hospital	Treviglio-Caravaggio hospital	Total
CTDIvol (mGy)	Q1	4.04	8.28	4.42
	Median	5.39	10.74	6.08
	Q3	6.58	13.82	8.84
	IQR	2.54	5.53	4.41
	Mean (SD)	5.58 (2.00)	11.41 (4.74)	7.29 (4.06)
DLP (mGy·cm)	Q1	158.75	282.64	171.75
	Median	208.50	382.00	237.50
	Q3	254.00	466.43	320.12
	IQR	95.25	183.79	148.38
	Mean (SD)	216.66 (74.40)	405.97 (201.69)	270.75 (151.02)

CTDIvol, volume CT dose index; DLP, dose-length product; IQR, interquartile range; SD, standard deviation

Overall, the results highlight the dual challenges of optimizing diagnostic yield and managing incidental findings within the framework of evidence-based guidelines. By identifying areas for improvement in both patient selection and guideline adherence, this study provides actionable insights into enhancing the sustainability and efficacy of radiological practice.

## Discussion

Findings from this multicentric retrospective study suggest that clearer preselection criteria could reduce low-yield examinations, especially in elective settings. With a diagnostic yield of 54.64%, the study highlights a potential overuse of chest CT in certain clinical scenarios, particularly in elective imaging settings where careful patient evaluation might reduce unnecessary imaging. This aligns with the broader objective of optimizing the balance between diagnostic efficacy and patient safety, particularly given the ionizing radiation exposure inherent in CT examinations.

Variability in diagnostic yield across different clinical queries was notable, ranging from 0% for pneumothorax (0 out of 10 cases, CI 0.00–30.85) to 80% for bronchiectasis (8 out of 10 cases, CI 49.03–94.33) and 75% for chronic respiratory conditions (36 out of 48 cases, CI 61.01–85.25). For conditions like pneumothorax and unexplained dyspnea (4 out of 18 cases, 22.22%, CI 8.48–45.23), alternative management strategies or enhanced clinical assessments could mitigate unnecessary radiation exposure and optimize resource allocation. Conversely, high-yield scenarios, such as bronchiectasis and chronic respiratory conditions, reaffirm the indispensable role of CT as a diagnostic cornerstone.

The incidence of incidental findings in our study (34.64%) closely mirrors the prevalence reported in comprehensive reviews and meta-analyses of chest CT imaging. Lumbreras et al. identified a rate of approximately 30% in thoracic CT, consistent with routine clinical observations [12]. O’Sullivan et al., in their analysis of elective imaging, reported similar figures, further validating these findings as a characteristic feature of diagnostic chest CT [13]. Meta-analytic data by Evans et al. highlighted an average prevalence of 33% across various CT modalities, including thoracic imaging in emergency contexts [14], while Davenport demonstrated analogous rates in non-urgent applications [15].

Such alignment with established literature emphasizes the ubiquity of incidental findings in chest CT and reinforces the critical need for standardized, evidence-based strategies to manage their clinical and operational implications effectively.

The study highlights significant variability in adherence to established guidelines for radiological findings management, with pulmonary findings showing the highest alignment (87.12%, CI 81.57–91.15). This strong adherence reflects the robustness and widespread adoption of lung-specific guidelines, particularly those from the Fleischner Society. In contrast, adherence rates for thyroid (42.11%, CI 24.86–61.84), liver (36.36%, CI 21.89–54.16), and kidneys (40.00%, CI 21.87–61.74) were substantially lower. These differences underscore critical gaps in standardization and highlight the need for dedicated educational initiatives and clinical protocols tailored to these regions.

Adherence for ‘other’ findings (89.47%, CI 75.96–95.72) was comparable to pulmonary findings, suggesting that established guideline frameworks effectively supported radiological decision-making in these cases. This category encompassed adrenal, pancreatic, and lymph node findings, where radiologists demonstrated a high degree of alignment with the recommendations proposed by the ACR, underscoring a consistent application of evidence-based criteria in these anatomical regions.

The observed variability emphasizes the importance of addressing non-pulmonary findings systematically, as current discrepancies could lead to inconsistent patient management. Developing or refining guidelines for non-pulmonary incidental findings, especially for regions lacking established protocols, could significantly enhance diagnostic precision and healthcare sustainability.

Moreover, while the inclusion of organs like the thyroid, liver, and kidneys in the imaging field varied, the study’s design reflects real-world practice, capturing an accurate snapshot of incidental finding prevalence. These results should guide future efforts to optimize guideline adherence across anatomical systems, ensuring comprehensive and equitable patient care.

Such standardization could reduce the inconsistencies observed in follow-up recommendations, where unnecessary imaging accounted for 14.29% of cases (CI 10.56–18.99) and under-recommendations for 4.28% (CI 2.43–7.47).

The implications of these findings extend beyond diagnostic accuracy to the broader realm of healthcare sustainability. The mean dose-length product (DLP) of 270.75 mGy·cm, while consistent with diagnostic reference levels, raises concerns about the cumulative radiation burden in patients undergoing unnecessary or non-evidence-based imaging. By refining preselection criteria and aligning practices with guidelines, radiology departments could significantly reduce this burden. Additionally, ensuring adherence to guidelines in managing radiological findings could prevent the cascade of unnecessary follow-ups, which not only contribute to patient anxiety but also strain healthcare resources.

This study also reflects the broader role of radiologists as gatekeepers in imaging pathways. Accurate reporting and appropriate recommendations are critical to ensuring that imaging contributes meaningfully to clinical decision-making while avoiding the pitfalls of overdiagnosis and overtreatment. The observed variability in guideline adherence, particularly in extra-thoracic regions, suggests a need for updated and universally accepted recommendations tailored to findings beyond the thorax. Incorporating these into routine workflows could enhance radiologists’ ability to make evidence-based recommendations.

As shown in Table 4, CTDIvol and DLP values were significantly higher at the Radiology Department of Treviglio-Caravaggio Hospital—ASST Bergamo Ovest than at the Radiology Unit of the Foundation IRCCS Ca’ Granda Ospedale Maggiore Policlinico of Milan, with nearly twofold differences in the medians and wider interquartile ranges. Although absolute exposure levels remained within accepted diagnostic reference thresholds [16, 17], the systematic variation observed between centers suggests scope for harmonization of acquisition protocols. The differences are likely attributable to local technical settings (e.g., tube current modulation, tube voltage, iterative reconstruction strength) and protocol preferences. Detailed scanner parameters were not collected, as dose optimisation was beyond the scope of this study; nevertheless, these findings underscore the importance of aligning acquisition strategies across institutions to reduce unwarranted variability while maintaining diagnostic consistency.

The strengths of this study are exemplified by its rigorous bicentric design, which enabled the collection of detailed and clinically representative data from two distinct institutions, and the deliberate inclusion of a meticulously defined cohort undergoing their first CT examination. These features provide a robust foundation for evaluating adherence to radiological guidelines and the management of findings within a real-world clinical framework. Nonetheless, several considerations warrant discussion, not as detractors from the study’s validity but as contextual nuances that enrich its interpretation and highlight areas for potential refinement in future investigations.

Firstly, the retrospective nature of the study, while inherently constrained by its reliance on historical data, introduces the possibility of selection biases. These risks were minimized through methodological rigor, including randomization and the distribution of imaging cases across the calendar year to mitigate the influence of seasonal variability. Secondly, although the study adhered to well-established guidelines from the Fleischner Society and the American College of Radiology (ACR), the transferability of these standards across diverse healthcare systems and clinical practices necessitates further exploration to ensure their global applicability and adaptability.

Additionally, the study does not incorporate downstream clinical outcomes following radiological recommendations, limiting insights into the broader implications of adherence to guidelines on patient trajectories and healthcare resource utilization. This omission highlights an avenue for future research to provide a more comprehensive assessment of the longitudinal impact of guideline-based imaging practices.

A particularly noteworthy consideration pertains to the heterogeneity in the anatomical regions included within the imaging field of view. The incidental visualization of structures beyond the thorax, such as the thyroid, liver, and kidneys, was not standardized across all scans. This variability likely influenced the frequency of incidental findings, which might have been underreported if these regions were infrequently captured, or overreported if consistently included. Importantly, this reflects the inherent variability of routine clinical practice, underscoring the real-world applicability of the study’s findings. Rather than a limitation in the strict sense, this aspect exemplifies the complexities radiologists encounter in daily practice, reinforcing the relevance of the study’s conclusions to contemporary imaging workflows.

By addressing these dimensions, this study not only underscores the critical role of adherence to evidence-based guidelines in optimizing diagnostic pathways but also provides a foundation for future investigations to explore how standardized practices can be harmonized across diverse clinical settings. Such endeavors have the potential to advance diagnostic precision, enhance the judicious allocation of healthcare resources, and promote the sustainability of radiological care in a rapidly evolving healthcare landscape.

In conclusion, this study highlights critical opportunities to refine imaging practices and enhance the sustainability of radiological care. By prioritizing evidence-based preselection criteria and promoting consistent adherence to guidelines, radiology departments can improve diagnostic efficacy, minimize unnecessary radiation exposure, and contribute to a more judicious allocation of healthcare resources. These findings advocate for a more resource-conscious approach to imaging, underpinned by the integration of robust, evidence-based guidelines into routine clinical workflows. Future research should explore the long-term clinical and economic impacts of these interventions to validate their potential for broader implementation.

## Data Availability

The datasets generated and analyzed during the current study are not publicly available due to institutional policies on data confidentiality but are available from the corresponding author on reasonable request.
